# *Mycobacterium tuberculosis* Beijing Genotype^1^

**DOI:** 10.3201/eid0912.030276

**Published:** 2003-12

**Authors:** Troels Lillebaek, Åse B. Andersen, Asger Dirksen, Judith R. Glynn, Kristin Kremer

**Affiliations:** * National Institute for Prevention and Control of Infectious Diseases and Congenital Disorders, Copenhagen, Denmark; † Rigshospitalet University Hospital, Copenhagen, Denmark; ‡ Gentofte University Hospital, Gentofte, Denmark; § London School of Hygiene and Tropical Medicine, London, United Kingdom; ¶ National Institute of Public Health and the Environment, Bilthoven, the Netherlands

**Keywords:** *Mycobacterium tuberculosis*, molecular epidemiology, genotype, Beijing family, W-strain, IS6110 RFLP

## Abstract

Molecular epidemiologic studies of strains of *Mycobacterium tuberculosis* are currently conducted worldwide. The genetically distinct Beijing family of strains has been associated with large outbreaks of tuberculosis, increased virulence, and multidrug resistance. However, in this first population-based search for Beijing strains in the Danish DNA fingerprint database, analysis of 97% of all culture-positive tuberculosis patients in 1992 to 2001, showed that 2.5% of 3,844 patients, 1.0% of Danish-born patients and 3.6% of immigrants (from 85 countries) had Beijing strains. No Beijing strains were found among 201 strains from Danish-born patients sampled in the 1960s, and no evidence of an increase in Beijing strains was found over time. The true prevalence of Beijing strains worldwide is unknown because only a fraction of global strains have been analyzed.

New technologies have enabled researchers to clarify fundamental questions about the epidemiology and pathogenesis of tuberculosis that were previously obscure (*1*). Although the *Mycobacterium tuberculosis* genome is genetically highly conserved, insertion sequences, repetitive elements, genomic deletions, and single nucleotide polymorphisms cause genetic polymorphisms. These polymorphisms can be visualized by various genotyping techniques, often referred to as DNA fingerprinting, whereby specific strains of *M. tuberculosis* can be characterized on the basis of their DNA patterns (*2*). Restriction fragment length polymorphism (RFLP) typing by using the insertion sequence IS*6110* as a probe for strain differentiation is the most widely applied DNA fingerprinting method to study the epidemiology of tuberculosis (*1*). This technique has been used for population-based transmission surveillance (*1*), including studies across national boundaries (*3*). In connection with this effort, one genetically highly conserved group of strains of *M. tuberculosis* collectively known as “the Beijing family” has attracted special attention (*2**,**4*). These strains are reported to be highly prevalent throughout Asia and in the countries of the former Soviet Union (*5*–*9*); they may possess selective advantages compared with strains of other *M. tuberculosis* genotypes (*5*); and they are sometimes associated with multidrug resistance (*6*,*8*,*10*,*11*) and with specific pathogenic properties and increased virulence (*6*,*8*,*12*). Furthermore, Beijing family strains may be increasing in frequency and be spreading to new geographic areas (*5*,*10*,*11*,*13*). The “W-strain family” concurrently identified on the North American (*10*) and Asian continents (*5*) is part of the Beijing family. In this study we investigated the Beijing strain family in Denmark.

## Methods

### Data Collection

Microbiologic analyses of mycobacteria have been carried out at the International Reference Laboratory of Mycobacteriology at Statens Serum Institut in Copenhagen since 1922. This is the only laboratory that performs culture-based tuberculosis diagnosis for the Danish Kingdom. Since 1992, DNA fingerprinting of strains of the *M. tuberculosis* complex has been implemented on a nationwide scale by using the internationally standardized method of IS*6110* RFLP typing (*14*). Fingerprints from a total of 4,102 strains from 3,844 patients were available for the current study, representing 97% of culture-positive patients in Denmark in 1992 to 2001. When more than one strain was available, the earliest specimen was included in the analysis. In addition, a search for Beijing family strains was performed among 201 strains of *M. tuberculosis* retrieved from tuberculosis patients from 1961 to 1967 (*15*). These strains were retrieved from Danish-born patients who were suspected of being part of various chains of local transmission. Ninety-five came from case-patients living in Copenhagen, the capital city, and its surroundings, where most new tuberculosis cases were, and still are, found. The strains were freeze-dried in the 1960s and recently recultured, and DNA fingerprinting was carried out (*16*,*17*). The strains were processed as previously described (*3*,*16*,*17*). The study was approved by the local medical ethics committees (No. 11-087/99) and the Danish Data Protection Agency (No. 2001-41-1018).

### Identification of Beijing Strains

Within the framework of the current European Union Concerted Action project (CA project), “New Generation Genetic Markers and Techniques for the Epidemiology and Control of Tuberculosis,” a method of identifying the Beijing family of strains by using IS*6110* RFLP typing was defined, on the basis of comparison with 19 reference strains (https://hypocrates.rivm.nl/bnwww/index.html) (K. Kremer et al., unpub. data). Following the CA project suggested methodology, strains of *M. tuberculosis* with IS*6110* patterns with >80% similarity to any of these strains could be classified as Beijing family strains, whereas strains showing 75% to 80% similarity needed to be confirmed by spoligotyping. This procedure should give a sensitivity of >98% and specificity of 100% for recognizing Beijing family strains (compared with the standard criterion of spoligotyping) (K. Kremer et al., unpub. data). For this study, for all strains showing at least 75% similarity to any of the reference strains, spoligotyping was used to confirm that they were indeed Beijing strains. For statistical analysis, the p values were calculated by the chi-square test or Fisher exact test when expected values were small.

## Results

Among the strains from the 1960s, no Beijing family strains were identified. The results from the more recent patients are summarized in Table 1. In total, 96 Beijing strains were retrieved from different patients. The spoligo patterns of 95 of these strains had 9 spacers and 1 strain (from a patient from Vietnam) had 7 spacers of the spacers 35 to 43. Overall, 56% of the tuberculosis patients were born outside of Denmark, originating from 85 different countries. Among Danish-born patients, 1.0% had Beijing strains compared to 3.6% among foreign-born patients (Table 1). The highest prevalence of Beijing strains was among patients from Asia. By country of origin the prevalence of the Beijing strain varied: 25.0% (24/96) from Vietnam, 33.3% (12/36) from Thailand, 0% (0/44) from the Philippines, 9.7% (3/31) from India, 8.8% (3/34) from Sri Lanka, and 0% (0/220) from Pakistan. Beijing strains were also found in 1.7% of patients from Somalia (17/985) and in patients from the Middle East, including 7.5% (3/40) from Iraq, 10.5% (2/19) from Iran, and 3.9% (1/ 26) from Afghanistan. No Beijing strains were found in patients from Eastern Europe: most of these patients (149) were from the former Yugoslavia; 6 were from the former Soviet Union.

**Table 1:** Proportion of tuberculosis patients with Beijing family strains^a^

	Denmark-born		Non–Denmark-born		Total	
	N/N (%)		N/N (%)		N/N (%)	
					
**All**	17/1,659 (1.0)		79/2,183 (3.6)		96/3,844 (2.5)	
					
Male	9/1,057 (0.85)		49/1,163 (4.2)		58/2,220 (2.6)	
Female	8/602 (1.3)		30/1018 (3.0)		38/1,620 (2.4)	
					
**Age group (y)**						
< 25	2/118 (1.7)		21/655 (3.2)		23/773 (3.0)	
25–44	7/553 (1.3)		48/1,159 (4.1)		55/1,712 (3.2)	
45–64	4/522 (0.77)		6/247 (2.4)		10/769 (1.3)	
65+	4/466 (0.86)		4/121 (3.3)		8/587 (1.4)	
					
**Y**						
1992–93	4/335 (1.2)		12/249 (4.8)		16/584 (2.7)	
1994–95	2/371 (0.54)		19/418 (4.6)		21/789 (2.7)	
1996–97	2/330 (0.61)		16/506 (3.2)		18/836 (2.2)	
1998–99	4/316 (1.3)		15/555 (2.7)		19/871 (2.2)	
2000–01	5/307 (1.6)		17/455 (3.7)		22/764 (2.9)	
					
**Area of origin**						
Western Europe	17/1,659 (1.0)		0/71 (0.0)		17/1,730 (0.98)	
Eastern Europe			0/174 (0.0)			
Indian subcontinent			8/290 (2.8)			
South East Asia			37/183 (20.2)			
East Asia and Pacific			3/10 (30.0)			
Middle East			6/211 (2.8)			
North Africa			1/38 (2.6)			
Sub–Saharan Africa			20/1,111 (1.8)			
Americas and Caribbean			0/16 (0.0)			
					
**Previous TB**						
No	17/1550 (1.1)		78/2164 (3.6)		95/3,716 (2.6)	
Yes	0/109 (0.0)		1/19 (5.3)		1/128 (0.79)	
					
**Site of TB**						
Pulmonary	16/1,394 (1.2)		56/1248 (4.5)		72/2,642 (2.7)	
Extrapulmonary	1/263 (0.38)		23/930 (2.2)		24/1,193 (2.0)	

^a^Information on immigration status missing for three patients; on region of origin for 81; on age for 3; on sex for 4; and on site of tuberculosis (TB) for 9.

No evidence was noted of an increase in the prevalence of Beijing strains over time. Although no Beijing strains were found in the 1960s, this finding is not significantly different from the prevalence among Danish patients in the recent period (p = 0.2). No increase occurred over the period of the current study from 1992 to 2001 among Danish patients or those born outside of Denmark (Table 1). An apparent trend towards an increased proportion of Beijing strains in younger patients seen overall (Table 1) is attributable to the higher proportion of immigrants in younger age groups. Only one of the patients with the Beijing strain had known previous tuberculosis (a patient from Somalia). Beijing strains were less common in those without pulmonary involvement (p = 0.007, adjusted for immigration). HIV status was not available for these patients.

The results of drug resistance testing are shown in Table 2. Among Danish patients, but not among immigrants, the infections of those who had Beijing strains were more likely to be drug resistant. The results, after excluding those with known previous tuberculosis, were very similar (not shown). Although some of these associations were formally statistically significant, they are based on only two drug-resistant cases among 16 Danish-born patients with Beijing strains.

**Table 2:** Proportion of patients with drug-resistant strains

	N	% Drug resistant (no. of patients with drug resistance)^a^						
		Any drug	Isoniazid	Rifampicin	Streptomycin	Pyrazinamide	Ethambutol	MDR^b^
								
**Danish**								
Beijing	16	12.5 (2)	12.5 (2)	6.3 (1)	12.5 (2)	0.0 (0)	6.3 (1)	6.3 (1)
Other	1,623	10.2 (165)	3.1 (50)	0.12 (2)	3.6 (58)	5.5 (89)	0.0 (0)	0.0 (0)
p		0.7	0.09	0.03	0.1	1.0	0.01	0.01
								
**Immigrants**								
Beijing	78	20.5 (16)	9.0 (7)	0 (0)	16.7 (13)	1.3 (1)	1.3 (1)	0.0 (0)
Other	2,086	17.2 (359)	7.5 (157)	0.72 (15)	13.6 (284)	1.3 (27)	0.96 (20)	0.58 (12)
p		0.4	0.7	1.0	0.4	1.0	0.5	1.0
								
**Overall**								
Beijing	94	19.2 (18)	9.6 (9)	1.1 (1)	16.0 (15)	1.1 (1)	2.1 (2)	1.1 (1)
**Other**	3,709	14.1 (524)	5.6 (207)	0.46 (17)	9.2 (342)	3.1 (116)	0.54 (20)	0.32 (12)
p		0.2	0.1	0.4	0.05	0.5	0.1	0.3

^a^Drug resistance data missing for 41 persons. ^b^MDR, multidrug resistant.

## Discussion

This population-based study found a low prevalence of Beijing strains and weak evidence of an association with drug resistance. The study includes an estimated 8% of all strains of *M. tuberculosis* IS*6110* RFLP typed worldwide from 1992 through 2001, of which 57% were retrieved from foreign-born patients from 85 different countries. Overall, only 2.5% of the patients had Beijing strains, and no evidence was found of an increase in their prevalence over time, even though Beijing strains have been found in Denmark for at least 10 years.

Recently, two studies analyzed the significance of *M. tuberculosis* transmission in Denmark due to immigration from a high incidence country and the persistent high incidence of tuberculosis among the immigrants in the years after arrival (*3*,*18*). These studies concluded that most (>75%) were infected before their arrival, that their latent infection was reactivated, and that nearly all of those who could have been infected after arrival (<23%) were most likely infected by a source from the country of origin (*3*). Therefore, in the present study we compared the observed prevalence with the prevalence in the country of origin. For example, 25% of patients from Vietnam had Beijing strains compared with 54% of patients in Hanoi and Ho Chi Minh City (*8*). However, the Vietnamese study included 563 samples from the late 1990s, whereas most Vietnamese-born immigrants arrived in Denmark during the early 1980s (*19*). This finding could indicate that Beijing strains have been emerging in Vietnam only since the early 1980s, which would fit with the higher prevalence of Beijing strains in persons of younger ages observed in the Vietnamese study. Regarding strains from patients born in Eastern Europe, none of the 174 patients had Beijing strains, compared with reports of 22% to 71% (*4*,*20*–*22*). However, the strains analyzed most were from patients from the former Yugoslavia, where the prevalence of Beijing strains is unknown. These patients arrived in Denmark during the 1990s. Our data suggest that the prevalence of Beijing strains was very low in this area, at least at that time. Few reports from Africa are available (*23*–*26*). In the present study, 17 (1.7%) of the 985 Somalia-born patients, nearly all of whom arrived in Denmark during the 1990s (*18*), had Beijing strains. Among the remaining 126 patients, who were born in 24 other African countries, three additional Beijing strains were retrieved, from patients born in Zimbabwe, Kenya, and Angola. Beijing strains seem to be rare on the African continent, but local studies are needed. Immigrants are not a random sample, and some may have acquired tuberculosis en route.

This is one of the largest samples of strains of *M. tuberculosis* searched for Beijing strains. Although highly representative for the Danish population in the 1990s, and partly for the Danish-born population in the 1960s, the IS*6110* RFLP patterns found in the strains from the foreign-born patients may not be an accurate reflection of the distribution of patterns in their country of origin. Also, identified patterns are a mixture of “recent” *M. tuberculosis* transmission and reactivation of latent infections and thus also represent patterns circulating decades ago (*16*,*17*).

The low prevalence we found contrasts with some reports, but limited information is available from most areas of the world, making definite conclusions about the extent of spread of Beijing strains and their associations with drug resistance premature (*4*). Studies in which Beijing strains have been looked for but not found may not have been published. Recently two studies from Delhi and Bombay, India, reported very few Beijing family strains (*27*,*28*). Similarly, both in this study and in a previous study, the prevalence of Beijing strains in the Phillippines was found to be very low, 0% and 2%, respectively (*29*). These findings indicate that even in Asia prevalence may show great variation. More unbiased studies, even those that report negative findings, are needed. However, the body of typing data is increasing, thereby disclosing a growing part of the true tuberculosis picture.
